# Non-prescription purchase of antibiotics during travel abroad among a general adult population in Norway: Findings from the seventh Tromsø Study

**DOI:** 10.1371/journal.pone.0228792

**Published:** 2020-02-13

**Authors:** Kirsten Gravningen, Nigel Field, Hege Salvesen Blix, Anne Mette Asfeldt, Lars Småbrekke

**Affiliations:** 1 Department of Microbiology and Infection Control, University Hospital of North Norway, Tromsø, Norway; 2 Centre for Molecular Epidemiology and Translational Research, Institute for Global Health, University College London, London, United Kingdom; 3 Department of Drug Statistics, Norwegian Institute of Public Health, Oslo, Norway; 4 Department of Pharmacy, University of Oslo, Oslo, Norway; 5 Department of Community Medicine, UiT - The Arctic University of Norway, Tromsø, Norway; 6 Department of Pharmacy, UiT - The Arctic University of Norway, Tromsø, Norway; University of Campania, ITALY

## Abstract

**Background:**

Non-prescription purchase of antibiotics is undesirable and has not recently been investigated in a representative population in a high-income low-use country during travel abroad. This study examined self-reported prevalence of antibiotic purchase abroad with and without prescription among participants reporting international travel in a general adult population in Norway, and the associations with socio-demographic, lifestyle and health factors.

**Methods:**

We analysed questionnaire-data from 19995 participants (10470 women) ≥40 years in the population-based Tromsø Study 7, 2015–2016. Data from the Norwegian Prescription Database were used to examine antibiotic use in Norway. We calculated adjusted odds ratios (AOR) for “travel abroad”, “any antibiotic purchase abroad”, and “antibiotic purchase abroad with” and “without prescription” using multivariable logistic regression.

**Results:**

Over half (55.0%, 95%CI 54.3–55.7%) participants reported travel abroad of >1 week duration in the past year. Travelers were more likely than non-travelers to be women (AOR = 2.02, 95%CI 1.42–2.88%) and report high education/income, childhood mostly lived abroad, healthy lifestyle, and good/excellent self-rated health. In total, 17904 travel episodes to 148 countries were reported. Altogether, 3.7% (95% CI 3.4%-4.1%) of travelers had purchased antibiotic abroad in the past year. Non-prescription purchase (1.5%, 95% CI 1.3–1.7) was associated with younger age, being female (AOR 1.41, 1.0–1.97), number of travels (reference: one episode, two: AOR = 1.82, 1.25–2.67, three: 2.60, 1.58–4.28, four: 3.10, 1.40–6.36 and ≥five: 4.70, 2.30–9.62), occurrences of diarrhoea (one: 2.42, 1.50–3.93 and ≥two: 3.08, 1.29–7.35), and antibiotic use in Norway in the past year (1.84, 1.29–2.62), whereas purchase with prescription (2.4%, 2.1–2.7) was associated with low income, growing-up abroad, recent hospital admission, additionally including number of travels/diarrhoea, and antibiotic use in Norway. Thailand (10.7%, 95% CI 7.8–14.3), Turkey (5.5%, 3.8–7.8) and Spain (3.6%, 3.0–4.3) were the countries most commonly associated with any antibiotic purchase. About two in five travelers who bought antibiotics in Thailand had done so without prescription, three in five in Turkey, and less than one in three in Spain.

**Conclusion:**

Overall, a small proportion of travelers had bought antibiotics abroad in the past year. Low prevalence of non-prescription purchase may be explained by awareness of the risks associated with self-medication, cultural views, unawareness of the non-prescription availability, and/or few infections. Divergent predictors for purchase abroad with versus without prescription may suggest different reasons for these practices.

## Introduction

Non-prescription supply of antibiotics is undesirable and remains high outside northern Europe and North America [1, 2]. More than 50% of antibiotics worldwide are obtained without a prescription, commonly in community pharmacies, and is particularly prevalent in low-income countries which do not enforce or do not have legislation restricting the sales and distribution of medicines [1, 3–5]. All the EU member states have legislation requiring that antibiotics are dispensed with prescription only, however, in 2016, an estimated 7% of antibiotics were taken without a prescription, most frequently in Romania (20%) and Greece (16%) [6]. Non-prescription use has been associated with shorter courses and inappropriate drug type and dosage [2, 7, 8]. Antibiotic use whether by prescription or not, is recognized as a major driver for antibiotic resistance both at the individual and population level and resistance is considered an increasingly important public health hazard worldwide [9–11]. In 2015, infections with selected antibiotic-resistant bacteria of public health importance accounted for over 33000 deaths in Europe [12].

Non-prescription availability has been associated with self-medication with antibiotics among people living in low-income countries [13]. To our knowledge, no recent representative study has investigated the purchase of antibiotics without prescription during travel abroad among people living in a high-income country with restrictive antibiotic policies. International travel is increasing and the number of international tourist arrivals worldwide doubled from 674 million in 2000 to 1326 million in 2017 [14]. People in Norway are among the world’s biggest travelers and popular travel destinations include countries where non-prescription sales of antibiotics is common, such as Spain, Greece and Thailand [2, 8, 15–20].

The antibiotic consumption in Norway is below the European average [11, 21] and an array of measures have been implemented to ensure effective antibiotic stewardship at a national level [10, 21]. Antibiotics have always been prescription-only medicines. There are antibiotic guidelines both for primary care and hospitals [22, 23], a surveillance system for outpatient antibiotic consumption [21], and laboratory-based surveillance of antibiotic resistance in bacteria [21]. In 2016, the government published an action plan on antibiotic resistance with the aim of reducing antibiotic use in the population by 30% by year-end 2020 compared with 2012. This includes public awareness campaigns and educational intervention to all general practitioners, and antibiotic sales had decreased by 24% in 2018 [24, 25].

Non-prescription antibiotic use in Norway is assumed to be very low. In the Eurobarometer survey 2016 on causes of non-prudent use of antibiotics in the EU (excluding Norway), 2% of people in Sweden and 6% in Denmark reported not having obtained their most recent antibiotic course from a medical practitioner [26]. It is likely that Norway has similar proportions.

The aims of this study were to examine the self-reported prevalence of antibiotic purchase abroad among participants reporting international travel in a general adult population in Norway, and how antibiotic purchase abroad was associated with socio-demographic, lifestyle and health factors among travelers. Both antibiotic purchase with and without prescription were studied.

## Materials and methods

### Study population and design

**Fig 1:**
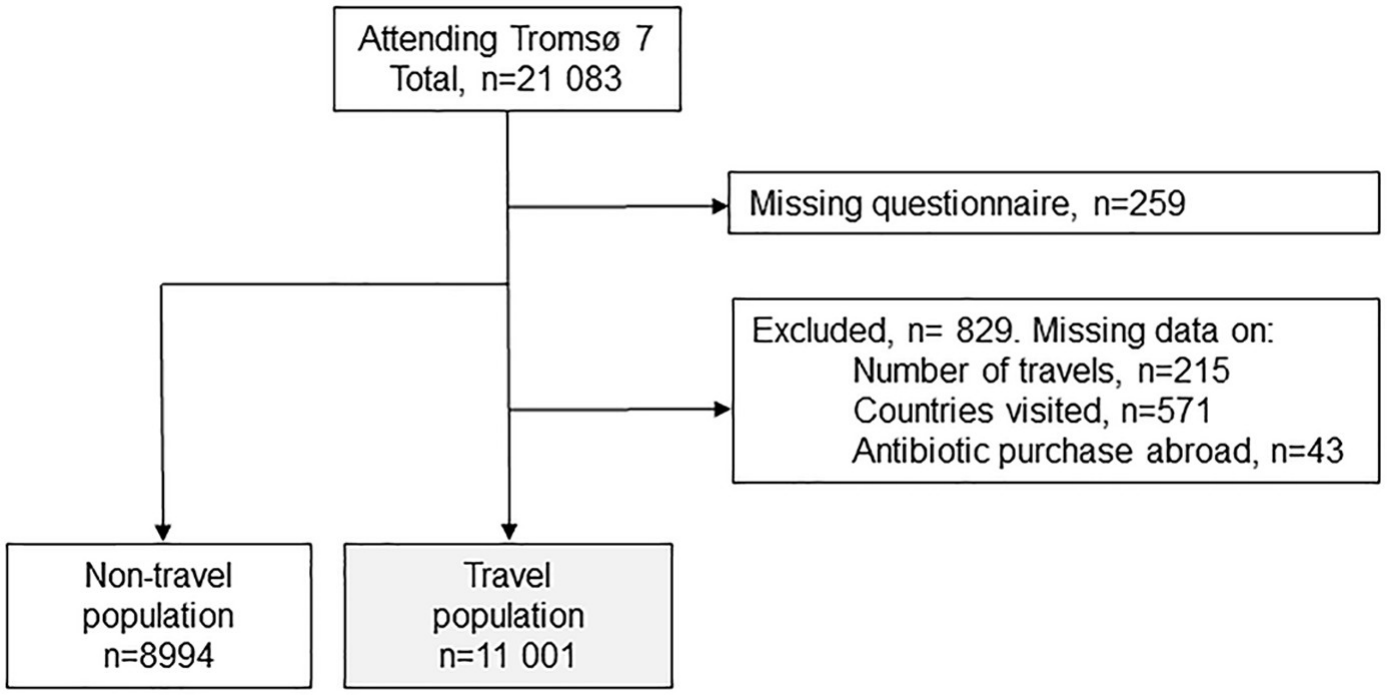
The study population. The seventh survey of the Tromsø Study.

The Tromsø Study is a longitudinal, population-based general health study in the municipality of Tromsø, Norway. Tromsø is considered as representative of a Northern European white, urban population and full details of the survey methods have been published elsewhere [27]. The seventh survey (Tromsø 7) was undertaken March 2015-October 2016 [28]. Unique national identity numbers from the official population-registry were used to invite all residents ≥40 years in Tromsø. The attendance rate was 64.7% (n = 21083). After excluding 1088 participants with missing questionnaire and/or non-response to key variables, we analysed cross-sectional questionnaire data from 19995 participants (10470 women) aged 40–89 years (Fig 1).

To assess participants’ antibiotic use in Norway the past 12 months, data from Tromsø 7 and the Norwegian Prescription Database (NorPD) were linked at the Norwegian Institute of Public Health [29]. NorPD includes detailed information at the individual level on all dispensed antibiotics at all pharmacies in Norway.

### Questionnaires and measurements

Participants in Tromsø 7 completed two self-administered structured questionnaires at home, either on paper or online [28] or, alternatively, at the first clinical screening visit where they could receive personal assistance. The questionnaires covered a broad range of issues related to sociodemographics, lifestyle and health, and sections for “travel and illness” and “antibiotics purchased abroad” (Box 1).

**Box 1. The questions used to assess “travel and illness” and “antibiotics purchased abroad”.**

**Travel and illness**

- Please, specify the number of travels you have performed during the past 12 months outside the Nordic countries with duration of >1 week. (Please, tick 0 if you had no travels of >1 week duration outside the Nordic countries in the past 12 months).
- For each travel outside the Nordic countries with duration of >1 week, please specify which country you stayed in—Travel 1 (to 20)? (A pull-down menu with 200 countries excluding the Nordic countries (i.e. Norway, Sweden, Denmark, Finland and Iceland) were displayed. Participants could select one country for each travel episode 1–20).
- For each travel outside the Nordic countries with a duration of >1 week, did you experience diarrhoea in connection with the travel—Travel episode 1 (-20). (No/yes)

**Antibiotics purchased abroad**

- Have you purchased antibiotics abroad during the last 12 months (penicillin-like medicine to treat infections)? (No/yes)
- If you have purchased antibiotics abroad during the last 12 months—how did you acquire it? Prescription from doctor/dentist? (No/yes)
- If you have purchased antibiotics abroad during the last 12 months—how did you acquire it? From a pharmacy, without a prescription? (No/yes)

“Travelers” included participants reporting travels outside the Nordic countries with duration >1 week the past 12 months. “Non-travelers” may thus include participants with travels of ≤1 week duration. Antibiotics dispensed (here defined as used) in Norway the past 12 months from NorPD included antibiotics for systemic use only (group J01 and P01AB01) in the Anatomical Therapeutic Chemical (ATC) classification system and are presented both as number of prescriptions dispensed and dichotomous variables [30]. A freely accessible web-tool, mapchart.net©, was used to create a world map where each country had a colour code corresponding to travel prevalence in the past year.

### Statistical analyses

SPSS v24.0 (NY: IBM Corp) was used for statistical analyses. We present descriptive characteristics by numbers and proportions (%) and calculated 95% confidence intervals (CIs) or standard deviation (SD) for means using student t-distribution, and 95% CIs for proportions using the exact binominal method. Logistic regression models were tested for a priori known or assumed related explanatory variables and only statistically significant variables were kept in the multivariable models together with the significant interaction terms with sex [13, 15]. We used logistic regression analysis to study the associations between selected explanatory variables and four outcome variables: 1) “travel” (travels of >1 week duration outside the Nordic countries past 12 months, no/yes, all participants), 2) “any antibiotic purchase abroad past 12 months” (no/yes, all travelers), 3) “antibiotic purchase abroad without prescription past 12 months” (no/yes, excluding travelers reporting “antibiotic purchase abroad with prescription”), and 4) “antibiotic purchase abroad with prescription past 12 months” (no/yes, excluding travelers reporting “antibiotic purchase abroad without prescription”). For all models, co-linearity was not a problem with variance inflation factor <1.25 for all variables. Two-sided p-values <0.05 were considered statistically significant. As the questions on antibiotic purchase abroad were asked after the”travel and illness”-section (Box 1) and not linked to each country visited, the countries associated with non-prescription antibiotic purchase were examined only among participants reporting travel to one country.

### Ethics

The study, including the linking of data between Tromsø 7 and NorPD, was approved by The Regional Committee for Medical and Health Research Ethics, North Norway (2016/1788/REK Nord) and complied with the Declaration of Helsinki. All participants in Tromsø 7 signed an informed consent declaration before participation.

## Results

### Characteristics of travelers

More than half of participants (55.0%, 95%CI: 54.3–55.7%, n = 11001) reported travel of >1 week duration in the past year outside the Nordic countries (Fig 1). Mean age for women travelers was 56.0 years (95%CI: 55.8–56.3) versus 58.3 years (57.9–58.6, p<0.001) for women non-travelers. The corresponding means for men were 56.8 years (56.5–57.1) and 57.9 years (57.5–58.2, p<0.001). In analyses adjusting for age, sex, household income, childhood mostly lived abroad and self-rated health, older age (≥80 years) was associated with less travel (adjusted odds ratio (AOR) 0.48) (Table 1).

**Table 1:** Characteristics of 19995 participants by international travel status in the past 12 months.

	No travel*	Travel							
	n = 8994	n = 11001	OR	95% CI	p-value	AORǂ	95% CI	p-value	*Denominators*†
**Age**									
40–49	30.1%	31.4%	1.00		<0.001	1.00		<0.001	*2703*, *3459*
50–59	27.0%	30.2%	1.07	1.00–1.15		1.10	1.02–1.19		*2426*, *3320*
60–69	23.4%	25.5%	1.04	0.97–1.12		1.21	1.01–1.33		*2102*, *2803*
70–79	14.1%	11.2%	0.76	0.69–0.83		1.10	0.97–1.26		*1267*, *1228*
80–95	5.5%	1.7%	0.30	0.25–0.36		0.48	0.39–0.61		*496*, *191*
**Sex**									
Men	46.1%	48.9%	1.00		<0.001	1.00		<0.001	*4150*, *5375*
Women	53.9%	51.1%	0.90	0.85–0.95		2.02	1.42–2.88		*4844*, *5626*
**Higher education level**^a^	41.1%	56.5%	1.86	1.75–1.97	<0.001	1.44	1.36–1.54	<0.001	*8779*, *10880*
**Higher household income**^b^	38.8%	56.9%	2.09	1.97–2.21	<0.001	1.79	1.64–1.96	<0.001	*8516*, *10691*
**Daily smoking**	17.2%	11.2%	0.61	0.56–0.66	<0.001	0.71	0.66–0.78	<0.001	*8901*, *10920*
**Alcohol intake ≥2 times/week**	22.3%	36.3%	1.99	1.87–2.12	<0.001	1.70	1.59–1.82	<0.001	*8924*, *10952*
**Living with spouse/cohabiting**	71.4%	81.2%	1.73	1.62–1.85	<0.001	1.21	1.11–1.31	<0.001	*8365*, *10540*
**Childhood mostly lived abroad**	2.8%	6.4%	2.37	2.04–2.74	<0.001	2.45	2.10–2.85	<0.001	*8958*, *10968*
**High physical activity**^c^	24.3%	30.0%	1.34	1.25–1.42	<0.001	1.16	1.08–1.24	<0.001	*8595*, *10754*
**Self-rated health good/excellent**	62.6%	73.9%	1.69	1.59–1.80	<0.001	1.50	1.40–1.60	<0.001	*8890*, *10939*
**Hospitalization past 12 months**	12.3%	9.5%	0.74	0.68–0.81	<0.001	0.85	0.77–0.94	0.001	*8893*, *10919*
**Chronic lung disease**^d^	3.2%	1.8%	0.54	0.45–0.65	<0.001	0.77	0.63–0.94	0.011	*8623*, *10699*
**Antibiotic use in Norway past 12 months**^e^	22.0%	21.0%	0.93	0.87–0.99	0.034	1.06	0.99–1.14	0.095	*8994*, *11001*

*No travel outside the Nordic countries >1 week duration the past 12 months.

OR, odds ratio (reference: No travel); CI, confidence interval; AOR, adjusted odds ratio; NA, not applicable.

^ǂ^AORs for age group were adjusted for sex, household income, childhood mostly lived abroad, self-rated health and statistically significant interaction terms. AORs for all other variables were adjusted for age as a continuous variable, sex, household income, childhood mostly lived abroad, self-rated health and statistically significant interaction terms.

^†^Denominators refer to number of participants included in the multivariable regression analyses of each independent variable (i.e. household income) or for a subgroup of each variable (i.e. age group 40-49y) and may vary due to missing information

^a^ ≥College/university degree

^b^ >751 000 NOK (€ 78 330/year as per July 2019)

^c^ Recreational sport >4 hours/week or hard training/sport competition several times a week

^d^ Chronic bronchitis/emphysema/COPD

^e^ Data from the Norwegian Prescription Database (NorPD)

Travelers were more likely than non-travelers to be women (AOR 2.02) and to report higher education level (AOR 1.44) and household income (1.79), more frequent alcohol intake (1.70), living with a spouse/partner (1.21), childhood mostly lived abroad (2.45), high physical activity (1.16) and good/excellent self-rated health (1.50) (Table 1). Travelers were less likely than non-travelers to report daily smoking (0.71), hospitalization past 12 months (0.85) and chronic lung disease (0.77). There was no statistically significant difference in the number of antibiotic prescriptions used in Norway the past 12 months between travelers (mean 0.47, SD 1.58) and non-travelers (0.38, 1.01).

### Travel destinations

**Fig 2:**
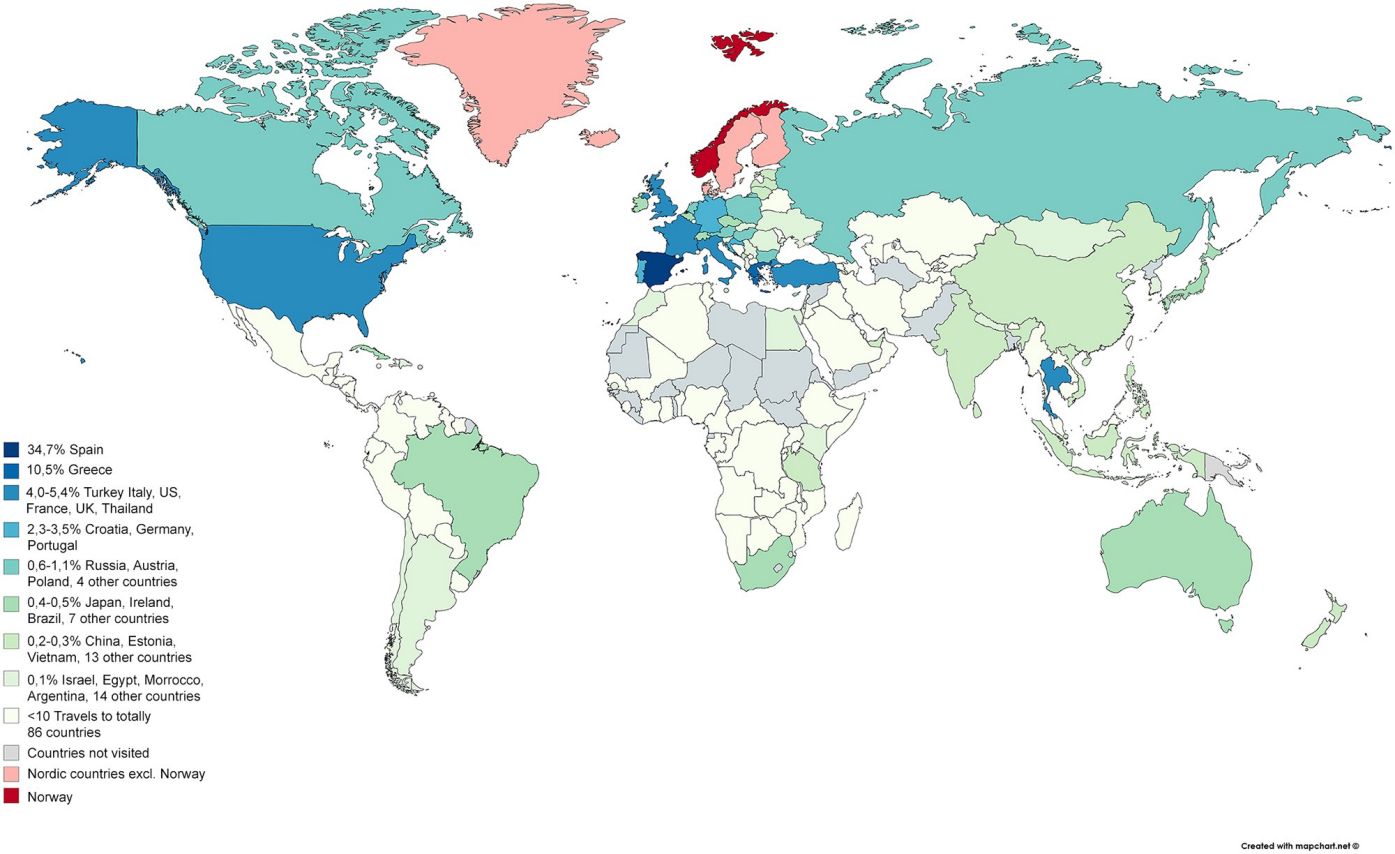
World map of travel destinations. Each country visited was assigned a colour code corresponding to travel prevalence in the past year.

11001 travelers reported a total of 17904 travel episodes to 148 countries in the past 12 months; 61% reported one travel, 26% reported two, 8% three, and 5% reported four travels or more. The countries most commonly visited were Spain 34.7%, Greece 10.5%, Turkey 5.4%, Italy 5.2%, US 4.9%, France 4.2%, UK 4.1% and Thailand 4.0% (Fig 2, S1 Table).

### Antibiotic purchase abroad

Altogether, 3.7% (95% CI 3.4%-4.1%, n = 410, no gender difference) of travelers reported purchase of antibiotics abroad in the past year (S2 Table). Antibiotic purchase abroad with prescription was reported by 2.4% (2.1–2.7, n = 262, no gender difference) of travelers and purchase without prescription by 1.5% (1.3–1.7, n = 170). A higher proportion of women (1.8%, 1.5–2.2) than men (1.3%, 1.0–1.6, p = 0.030) reported purchase without prescription. 22 travelers (13 women and 9 men) reported antibiotic purchase both with and without prescription.

Among non-travelers, 0.4% (0.3–0.6, n = 40) reported purchase of antibiotics abroad the past year; 0.3% (0.2–0.4, n = 26) with prescription and 0.2% (0.1–0.2, n = 14) without prescription.

In analyses of travelers only (adjusting for age, sex, number of travels abroad, and antibiotic use in Norway the past 12 months), younger participants (AOR = 0.97 per year, 0.96–0.99), women (1.41, 1.01–1.97) and those having used antibiotics in Norway (1.84, 1.29–2.62) were more likely to report purchase of non-prescription antibiotics abroad in the past year (Table 2).

**Table 2:** Factors associated with non-prescription and by-prescription purchase of antibiotics abroad among travelers.

	Non-prescription purchase*				By-prescription purchase**				*Denominators*¶
Characteristics	n = 149	AOR†	95% CI	p-value	n = 241	AORǂ	95% CI	p-value	*Non-prescript*., *by-prescription*
**Age** (continuous variable)	NA	0.97	0.96–0.99	0.001	NA	1.005	0.99–1.02	0.477	*10739*, *10831*
**Sex**									
Men	1.1%	1.00		0.042	2.4%	1.00		0.118	*5498*, *5525*
Women	1.6%	1.41	1.01–1.97		2.1%	0.81	0.62–1.06		*5241*, *5306*
**Education**									
<College/university degree	1.2%	1.00		0.891	2.6%	1.00		0.097	*4610*, *4675*
≥College/university degree	1.5%	1.02	0.73–1.45		2.0%	0.79	0.60–1.04		*6011*, *6036*
**Household income**									
Higher	1.5%	1.00		0.935	1.7%	1.00		0.001	*5979*, *5991*
Lower	1.3%	0.99	0.70–1.40		2.9%	1.63	1.22–2.17		*4460*, *4532*
**Current daily smoking**									
No	1.4%	1.00		0.665	2.3%	1.00		0.156	*9465*, *9551*
Yes	1.5%	1.12	0.68–1.84		1.8%	0.70	0.43–1.14		*1195*, *1199*
**Alcohol intake**									
< 2–3 times a week	1.2%	1.00		0.074	2.3%	1.00		0.399	*6800*, *6875*
≥ 2 times a week	1.7%	1.36	0.97–1.89		2.1%	0.81	0.51–1.27		*3890*, *3907*
**Childhood mostly lived abroad**									
No	1.4%	1.00		0.790	2.1%	1.00		0.041	*10034*, *10112*
Yes	1.9%	1.08	0.60–1.95		3.8%	1.64	1.05–2.57		*674*, *687*
**Hospital admission past 12m**									
No	1.4%	1.00		0.775	2.0%	1.00		0.002	*9668*, *9732*
Yes	1.5%	1.08	0.63–1.87		4.0%	1.76	1.23–2.51		*992*,*1018*
**Chronic lung disease** ^a^									
No	1.4%	NA		NA	2.1%	1.00		0.094	*10274*, *10346*
Yes	0.0%	NA	NA		5.4%	1.82	0.90–3.69		*176*, *186*
**Number of travels past 12m**									
1 travel	1.0%	1.00		<0.001	1.6%	1.00		<0.001	*6560*, *6601*
2 travels	1.7%	1.82	1.25–2.67		2.8%	1.80	1.32–2.45		*2757*, *2788*
3 travels	2.3%	2.60	1.58–4.28		3.9%	2.66	1.79–3.96		*894*, *908*
4 travels	2.7%	3.10	1.40–6.36		4.9%	3.08	1.70–5.60		*299*, *306*
≥ 5 travels	3.9%	4.70	2.30–9.62		3.5%	2.28	1.08–4.81		*229*, *228*
**Diarrhoea during travel past 12m**									
No	1.2%	1.00		<0.001	2.0%	1.00		<0.001	*9925*, *10001*
Yes, during one travel	3.1%	2.42	1.50–3.93		5.6%	2.72	1.86–3.97		*644*, *661*
Yes, during two or more travels	6.3%	3.08	1.29–7.35		6.3%	1.73	0.68–4.41		*96*, *96*
**Antibiotic use in Norway past y**^b^									
No	1.2%	1.00		<0.001	1.8%	1.00		<0.001	*8529*, *8582*
Yes	2.1%	1.84	1.29–2.62		3.8%	2.18	1.65–2.89		*2210*, *2249*

OR, odds ratio; CI, confidence interval; AOR, adjusted odds ratio; NA, not applicable; m, months

*Study population: 10739 travelers (i.e. excluding 262 travelers who purchased antibiotics abroad *with* prescription)

**Study population: 10831 travelers (i.e. excluding 170 travelers who purchased antibiotics abroad *without* prescription)

^†^Adjusted for age, sex, number of travels abroad past 12 months, and purchase of antibiotics in Norway.

^**ǂ**^ Adjusted for age, sex, household income, childhood mostly lived abroad, hospital admission, number of travels abroad past 12 months, and purchase of antibiotics in Norway.

^¶^ Denominators refer to number of participants included in the multivariable regression analyses for each independent variable (i.e. household income), or for a subgroup of each variable (i.e. 2 travels) and may vary due to missing information.

^a^ Chronic bronchitis/emphysema/COPD

^b^ Data from the Norwegian Prescription Database

A strong dose-response association was observed for increasing number of travel episodes and occurrences of diarrhoea during travel.

In contrast (in analyses adjusting for age, sex, income, childhood mostly lived abroad, recent hospital admission, number of travels abroad, and antibiotic use in Norway the past 12 months), antibiotic purchase abroad with prescription in the past year among travelers was associated with lower income (1.63, 1.22–2.17), childhood mostly lived abroad (1.64, 1.05–2.57) and recent hospital admission (1.76, 1.23–2.51), also including a dose-response association for increasing number of travel episodes and occurrences of diarrhoea, and antibiotic use in Norway (2.18, 1.65–2.89) (Table 2).

### Antibiotic purchase abroad and travel destination

Among 8159 of 11001 (74%) of travelers reporting travel to one country only, 104 reported purchase of antibiotics abroad without prescription and 176 reported purchase with prescription. The three countries most commonly associated with any purchase of antibiotics were Thailand (10.7%, 95% CI 7.8%-14.3%), Turkey (5.5%, 3.8–7.8) and Spain (3.6%, 3.0–4.3) (Table 3).

**Table 3:** Any purchase of antibiotics abroad vs. proportion of non-prescription purchase abroad by country, among 8159 travelers who visited one country only in the past year.

Travel destination	Number of travelers	Any antibiotic purchase abroad*			Non-prescription purchase†		
		(n)	%	95% CI	(n)	%	95% CI
**Thailand**	382	41	10.7%	7.8–14.3	17	42.5%	26.3–57.9
**Turkey**	544	30	5.5%	3.8–7.8	18	60.0%	40.6–77.3
**Spain**	3227	116	3.6%	3.0–4.3	36	31.0%	22.8–40.3
**France**	275	9	3.3%	1.5–6.1	2	25.0%	2.8–60.0
**Portugal**	158	4	2.5%	0.7–6.4	2	50.0%	6.8–93.2
**Italy**	373	7	1.9%	0.8–3.8	4	57.1%	18.4–90.1
**Greece**	1106	20	1.8%	1.1–2.8	8	40.0%	19.1–63.9
**More than one country**^a^	2842	151	5.3%	4.5–6.2	66	43.7%	35.7–52.0
**One country only**^b^	8159	277	3.4%	3.0–3.8	104	37.5%	31.8–43.5

CI, confidence interval

*In the past year

^†^Non-prescription purchase abroad as a proportion of any antibiotic purchase abroad in the past year

^a^ Travelers having visited *more than one country* in the past year (n = 2842)

^b^ Travelers having visited only one country in the past year (n = 8149)

Mean prevalence of any purchase was 3.4% (3.0–3.8) among travelers having visited one country only in the past year, and 5.3% (4.5–6.2) among those having visited more than one country. About two in five travelers who had bought antibiotics in Thailand did so without prescription, three in five in Turkey, and less than one in three in Spain. The proportion of non-prescription purchase was 37.5% (31.8–43.5) among travelers having visited one country only and 43.7% (35.7–52.0) among those having been to more than one country. Purchase of antibiotics both with and without prescription among 22 travelers, was most frequently associated with having visited Spain (n = 6), Turkey (n = 3) and Thailand (n = 3).

## Discussion

This large representative population study confirmed that adult people in Norway are frequent international travelers and that those who travel abroad are more likely to report high socioeconomic status, healthy behaviours, good health and having lived mostly abroad during childhood. Overall, a small proportion of travelers had bought antibiotics abroad in the past year. A small minority reported non-prescription purchase. Both by-prescription and non-prescription purchase had a strong dose-response relationship with number of travels and diarrhoea and were associated with antibiotic use in Norway in the past year. Non-prescription purchase was more likely among women and younger travelers, whereas by-prescription purchase was associated with low income, growing-up abroad and recent hospital admission, suggesting different reasons for these practices. Thailand, Turkey and Spain were the travel destinations most commonly associated with any antibiotic purchase abroad and the proportion of non-prescription purchase varied between countries.

### Strengths and limitations

To our knowledge, this is the first study of international travelers from a high-income country investigating their purchase of antibiotics abroad both with and without prescription, and relating this to national registry data on antibiotic use in their home country. An important methodological strength underlying our findings is that we used data from a representative sample of the Norwegian population of around 20,000 people aged ≥40 years in Tromsø 7, thereby avoiding the substantial selection bias related to sampling online, in travel clinics or at other convenience venues. A major strength also includes the high attendance rate. Despite this, the very low proportion reporting non-prescription purchase of antibiotics abroad hampered the statistical power of some subgroup analyses.

The age restricted sample is a limitation as antibiotic use in the population in Norway varies with age and is higher among older people [21]. Unfortunately, the participants were not asked about the purpose of their trip, such as holiday, work or study, and only travels longer than one week were included. However, very few in the non-travel group reported antibiotic purchase abroad, indicating that this rarely occurred during shorter travels. It is a limitation that purchase of antibiotics abroad was self-reported and thus prone to bias due to poor recall, social-desirability and/or misclassification, however, this may be the only possible approach to obtaining this information. In an internet survey in Norway in 2015, 97% of 11,500 respondents answered that antibiotics are medicines used for bacterial infections, indicating that people in Norway know what antibiotics are and therefore, we assume that misclassification bias in our study was low [31]. We recognize the limitations that it was not asked about the type of antibiotic being purchased, reasons for taking it, if participants had brought their own antibiotics from home, participants’ country of origin, and that only one country could be reported for each travel episode. Finally, the questions on travel destinations and purchase of antibiotics abroad were not directly linked and, therefore, only associations between country and purchase could be inferred.

### Comparison with other studies

The high prevalence of international travel among adults in Norway may be due to the relatively high income, low unemployment rate, high living costs, and the long cold winters, all of which could make travel to cheaper foreign destinations more attractive [17]. Our findings that people with high income and education were more likely to report travel abroad correspond with other studies as this group has the resources and opportunities to travel [15]. In line with this, travelers had a favorable health-risk profile, including nonsmoking and high physical activity [32, 33]. Furthermore, people of higher socioeconomic status are more likely to consume alcohol and tend to drink more frequently than those of lower status, as also observed among the travelers in our study [34]. The strong association between having lived mostly abroad during childhood and international travel in adult life could partly be due to migrants going back to visit their country of origin [35].

Although participants had travelled extensively to countries where non-prescription antibiotic purchase is possible, only a small proportion had used the opportunity. This desired behaviour may be explained by awareness of the risks associated with self-medication and/or unawareness of the non-prescription availability [13, 26]. However, purchase with prescription was also rare which could be due to travelers’ good health/few infections, previous experience of self-limiting infections, distrust in medication abroad, lack of information on where to get medication, and/or knowledge that antibiotics do not cure viral infections. Cultural views of which conditions that need antibiotic treatment may also have influenced the low overall low use [36]. The Eurobarometer survey 2016 found that over-the-counter selling in pharmacies is among the main causes for non-prescription antibiotic use and that those having been exposed to information on prudent use were more knowledgeable about the subject and more likely to obtain antibiotics from a medical practitioner [26]. However, this survey did not assess non-prescription purchase of antibiotics during travel abroad. A population survey in Denmark in 2003 found that 1% reported non-prescription purchase during travel abroad the past 12 months, suggesting that despite the boom in international travel this practice has remained low among people in Scandinavia [37].

The considerable dose-response association observed between both non-prescription and by-prescription purchase and number of travels in our study suggests that, although prevalence was low, increased exposure is a strong determinant for purchase among travelers from countries with strictly regulated antibiotic sales. In line with this, a study among immigrants from low-use country Finland, living permanently in Spain, found that 40% had purchased non-prescription antibiotics for common flu the past 6 months [38].

The strong association between diarrhoea during travel and purchase of antibiotics abroad detected in our study was also found in a Dutch travel study where half of those who had used antibiotics to cure diarrhoea had bought it locally over-the-counter [39]. Norwegian guidelines do not recommend prophylactic prescription of antibiotics to travelers [22].

Among the most frequently visited destinations, non-prescription purchase of antibiotics abroad was most commonly reported by travelers to Thailand and Turkey where multiple types of antibiotics are easily available in pharmacies without prescription [20, 40].

### Implications for practice and future research

Future research on this topic should ask about the purpose and length of the trip, country of purchase, antibiotic type, reasons for taking it, bringing antibiotics from home, participants’ country of origin, and address other sources for self-medication. People younger than 40 years should be included. However, one may argue that research rather should be conducted in countries where non-prescription sales are more prevalent. There is a need for well-designed studies on non-prescription supply of antibiotics to identify how level of enforcement of legislation is associated to non-prescription use and develop policy options aimed at strengthening prudent antibiotic use [6].

## Conclusions

Overall, a small proportion of travelers had bought antibiotics abroad. Low prevalence of non-prescription purchase may reflect little need for antibiotics during travel, awareness of the risks associated with self-medication, cultural views, unawareness of the non-prescription availability, and/or the longstanding tradition of low antibiotic use in Norway.

## Supporting information

S1 Table The 148 countries visited and number of travels (by country) among 11001 participants reporting travel >1 week duration outside the Nordic countries in the past year. (DOCX)

S2 Table Factors associated with any antibiotic purchase abroad among 11001 participants reporting international travel in the past year. (DOCX)

## References

[1] Auta A, Hadi MA, Oga E, Adewuyi EO, Abdu-Aguye SN, Adeloye D, et al Global access to antibiotics without prescription in community pharmacies: A systematic review and meta-analysis. J Infect. 2019;78(1):8–18. 10.1016/j.jinf.2018.07.001 29981773

[2] Morgan DJ, Okeke IN, Laxminarayan R, Perencevich EN, Weisenberg S. Non-prescription antimicrobial use worldwide: a systematic review. Lancet Infect Dis. 2011;11(9):692–701. 10.1016/S1473-3099(11)70054-8 21659004 PMC3543997

[3] Cars O, Nordberg P. Antibiotic resistance—The faceless threat. International Journal of Risk & Safety in Medicine. 2005;17(3):103–10.

[4] Hadi MA, Karami NA, Al-Muwalid AS, Al-Otabi A, Al-Subahi E, Bamomen A, et al Community pharmacists’ knowledge, attitude, and practices towards dispensing antibiotics without prescription (DAwP): a cross-sectional survey in Makkah Province, Saudi Arabia. Int J Infect Dis. 2016;47:95–100. 10.1016/j.ijid.2016.06.003 27343987

[5] Ocan M, Obuku EA, Bwanga F, Akena D, Richard S, Ogwal-Okeng J, et al Household antimicrobial self-medication: a systematic review and meta-analysis of the burden, risk factors and outcomes in developing countries. BMC public health. 2015;15:742 10.1186/s12889-015-2109-3 26231758 PMC4522083

[6] NIVEL and University of Antwerp (NL) for the European Commission. Antimicrobial Resistance and causes of Non-prudent use of Antibiotics in human medicine in the EU (The ARNA project) Brussels: European Union; 2017 [https://ec.europa.eu/newsroom/sante/newsletter-specific-archive-issue.cfm?newsletter_service_id=327&newsletter_issue_id=4487].

[7] Awad A, Eltayeb I, Matowe L, Thalib L. Self-medication with antibiotics and antimalarials in the community of Khartoum State, Sudan. J Pharm Pharm Sci. 2005;8(2):326–31. 16124943

[8] Saengcharoen W, Lerkiatbundit S. Practice and attitudes regarding the management of childhood diarrhoea among pharmacies in Thailand. Int J Pharm Pract. 2010;18(6):323–31. 10.1111/j.2042-7174.2010.00066.x 21054592

[9] Costelloe C, Metcalfe C, Lovering A, Mant D, Hay AD. Effect of antibiotic prescribing in primary care on antimicrobial resistance in individual patients: systematic review and meta-analysis. BMJ. 2010;340:c2096 10.1136/bmj.c2096 20483949

[10] Goossens H, Ferech M, Vander Stichele R, Elseviers M. Outpatient antibiotic use in Europe and association with resistance: a cross-national database study. Lancet. 2005;365(9459):579–87. 15708101 10.1016/S0140-6736(05)17907-0

[11] European Centre for Disease Prevention and Control (ECDC). Antimicrobial consumption. In: ECDC. Annual Epidemiological report for 2017. Stockholm; 2018 [https://ecdc.europa.eu/sites/portal/files/documents/ESAC-NET-reportAER-2017-updated.pdf.]

[12] Cassini A, Hogberg LD, Plachouras D, Quattrocchi A, Hoxha A, Simonsen GS, et al Attributable deaths and disability-adjusted life-years caused by infections with antibiotic-resistant bacteria in the EU and the European Economic Area in 2015: a population-level modelling analysis. Lancet Infect Dis. 2019;19(1):56–66. 10.1016/S1473-3099(18)30605-4 30409683 PMC6300481

[13] Grigoryan L, Burgerhof JG, Degener JE, Deschepper R, Lundborg CS, Monnet DL, et al Determinants of self-medication with antibiotics in Europe: the impact of beliefs, country wealth and the healthcare system. J Antimicrob Chemother. 2008;61(5):1172–9. 10.1093/jac/dkn054 18296694

[14] United Nations World Tourism Organization (UNWTO). UNWTO Tourism Highlights 2017 Geneva, Switzerland; 2018 [https://www.e-unwto.org/doi/pdf/10.18111/9789284419876.]

[15] Statistics Norway (SSB). Travel survey, fourth quarter of 2016. Source Tables 04464, 05717 and 06921 2017 [https://www.ssb.no/reise.]

[16] Institute of Transport Economics & Norwegian Centre for Transport Research. The National Travel Survey 2013/2014—key report. TØI report 1383/2014 Oslo 2014 [https://www.toi.no/getfile.php/1339511/Publikasjoner/T%C3%98I%20rapporter/2014/1383-2014/1383-2014-elektronisk.pdf].

[17] World Atlas RTI. World Facts: Countries That Travel The Most [updated March 15, 2019. https://www.worldatlas.com/articles/countries-whose-citizens-travel-the-most.html.]

[18] Guinovart MC, Figueras A, Llor C. Selling antimicrobials without prescription—Far beyond an administrative problem. Enferm Infecc Microbiol Clin. 2018;36(5):290–2. 10.1016/j.eimc.2016.10.006 27866752

[19] Plachouras D, Kavatha D, Antoniadou A, Giannitsioti E, Poulakou G, Kanellakopoulou K, et al Dispensing of antibiotics without prescription in Greece, 2008: another link in the antibiotic resistance chain. Euro Surveill. 2010;15(7). 20184852

[20] Sommanustweechai A, Chanvatik S, Sermsinsiri V, Sivilaikul S, Patcharanarumol W, Yeung S, et al Antibiotic distribution channels in Thailand: results of key-informant interviews, reviews of drug regulations and database searches. Bull World Health Organ. 2018;96(2):101–9. 10.2471/BLT.17.199679 29403113 PMC5791780

[21] NORM/NORM-VET 2018. Usage of Antimicrobial Agents and Occurrence of Antimicrobial Resistance in Norway. Norwegian Surveillance System for Antimicrobial Resistance. 2019 [https://unn.no/fag-og-forskning/norm-norsk-overvakingssystem-for-antibiotikaresistens-hos-mikrober#rapporter.]

[22] National guidelines for antibiotic use in primary care: Norwegian Directorate of Health; 2012. Last update Dec 11, 2018. [cited 2019 February]. https://helsedirektoratet.no/retningslinjer/nasjonal-faglig-retningslinje-for-antibiotikabruk-i-primerhelsetjenesten.

[23] Norwegian guideline for antibiotic use in hospitals: Norwegian Directorate of Health; 2015 [cited 2019 February]. https://helsedirektoratet.no/retningslinjer/antibiotika-i-sykehus.

[24] Ministry of Health and Care Services. National Action Plan to prevent Antimicrobial Resistance in Healthcare. Oslo; 2016 [https://www.regjeringen.no/no/dokumenter/handlingsplan-mot-antibiotikaresistens-i-helsetjenesten/id2469646/.]

[25] Antibiotic Centre for Primary Care—University of Oslo. ENORM—Educational intervention in Norwegian Municipalities for antibiotic treatment in line with guidelines; 2016 [https://www.med.uio.no/helsam/forskning/prosjekter/rak/].

[26] European Commission. Special Eurobarometer 445—April 2016 "Antimicrobial Resistance" 2016. [https://ec.europa.eu/health/amr/sites/amr/files/eb445_amr_generalreport_en.pdf]

[27] Jacobsen BK, Eggen AE, Mathiesen EB, Wilsgaard T, Njolstad I. Cohort profile: the Tromso Study. Int J Epidemiol. 2012;41(4):961–7. 10.1093/ije/dyr049 21422063 PMC3429870

[28] UiT—the Arctic University of Tromsø. Web page with questionnaires Q1 and Q2 for The seventh survey of the Tromsø Study (Tromsø 7) Tromsø, Norway. 2016 [https://uit.no/forskning/forskningsgrupper/sub?sub_id=503778&p_document_id=367276].

[29] Norwegian Institute of Public Health (NIPH). Norwegian Prescription Database, NorPD [cited 2019 February]. https://www.fhi.no/en/hn/health-registries/norpd/norwegian-prescription-database/.

[30] WHO Collaborating Centre for Drug Statistics Methodology. International language for drug utilization research National Institute of Public Health (NIPH), Oslo [cited 2018 August]. https://www.whocc.no/ddd/definition_and_general_considera/.

[31] Norwegian Intitute of Public Health (NIPH). Report from an Internet-survey on antmicrobial resistance Oslo 2015 [cited 2019 August]. https://www.fhi.no/globalassets/dokumenterfiler/moba/pdf/rapport-fra-nettbasert-sporreundersokelse-om-antibiotikaresistens-2015.pdf.pdf.

[32] Jha P, Peto R, Zatonski W, Boreham J, Jarvis MJ, Lopez AD. Social inequalities in male mortality, and in male mortality from smoking: indirect estimation from national death rates in England and Wales, Poland, and North America. Lancet. 2006;368(9533):367–70. 10.1016/S0140-6736(06)68975-7 16876664

[33] Allen L, Williams J, Townsend N, Mikkelsen B, Roberts N, Foster C, et al Socioeconomic status and non-communicable disease behavioural risk factors in low-income and lower-middle-income countries: a systematic review. Lancet Glob Health. 2017;5(3):e277–e89. 28193397 10.1016/S2214-109X(17)30058-X PMC5673683

[34] Bloomfield K, Grittner U, Kramer S, Gmel G. Social inequalities in alcohol consumption and alcohol-related problems in the study countries of the EU concerted action ‘Gender, Culture and Alcohol Problems: a Multi-national Study'. Alcohol Alcohol Suppl. 2006;41(1):i26–36. 10.1093/alcalc/agl073 17030500

[35] Aubry C, Gaudart J, Gaillard C, Delmont J, Parola P, Brouqui P, et al Demographics, health and travel characteristics of international travellers at a pre-travel clinic in Marseille, France. Travel Med Infect Dis. 2012;10(5–6):247–56. 10.1016/j.tmaid.2012.09.004 23062668

[36] Borg MA. National cultural dimensions as drivers of inappropriate ambulatory care consumption of antibiotics in Europe and their relevance to awareness campaigns. J Antimicrob Chemother. 2012;67(3):763–7. 10.1093/jac/dkr541 22200725

[37] Muscat M, Monnet DL, Klemmensen T, Grigoryan L, Jensen MH, Andersen M, et al Patterns of antibiotic use in the community in Denmark. Scand J Infect Dis. 2006;38(8):597–603. 10.1080/00365540600606507 16857602

[38] Vaananen MH, Pietila K, Airaksinen M. Self-medication with antibiotics—does it really happen in Europe? Health policy. 2006;77(2):166–71. 10.1016/j.healthpol.2005.07.001 16095749

[39] Belderok SM, van den Hoek A, Kint JA, Schim van der Loeff MF, Sonder GJ. Incidence, risk factors and treatment of diarrhoea among Dutch travellers: reasons not to routinely prescribe antibiotics. BMC Infect Dis. 2011;11:295 10.1186/1471-2334-11-295 22035314 PMC3223148

[40] Okuyan B, Savan MA, Izzettin FV, Sancar M. Evaluation of Rational Antibiotic Dispensing in the Community Pharmacy Setting: A Simulated Patient Study. Acta Pharm Sci. 2017;55(2):7–16.

