# Supplementary material for: Non-prescription purchase of antibiotics during travel abroad among a general adult population in Norway: Findings from the seventh Tromsø Study
Source: PLoS One. 2020 Feb 13;15(2):e0228792. doi: 10.1371/journal.pone.0228792 (PMC7017991; doi:10.1371/journal.pone.0228792)
Supplement: S1 Table — (DOCX) [file pone.0228792.s001.docx]

**Supplementary Table 1.**

| **Countries visited (n=148)** | **Number of travels to each country past y** | **Proportion (%) of total number of travels** |
| --- | --- | --- |
| Spain | 6212 | 34.7% |
| Greece | 1885 | 10.5% |
| Turkey | 971 | 5.4% |
| Italy | 931 | 5.2% |
| US | 878 | 4.9% |
| France | 752 | 4.2% |
| United Kingdom | 728 | 4.1% |
| Thailand | 710 | 4.0% |
| Croatia | 623 | 3.5% |
| Germany | 603 | 3.4% |
| Portugal | 411 | 2.3% |
| Russian Federation | 199 | 1.1% |
| Austria | 191 | 1.1% |
| Poland | 177 | 1.0% |
| Bulgaria | 163 | 0.9% |
| Netherlands | 143 | 0.8% |
| Canada | 113 | 0.6% |
| Hungary | 104 | 0.6% |
| Japan | 91 | 0.5% |
| Ireland | 89 | 0.5% |
| Brazil | 86 | 0.5% |
| Switzerland, Cyprus | 81 | 1.0% |
| South Africa, Australia | 77 | 0.8% |
| Czech Republic | 65 | 0.4% |
| Belgium, Cuba | 64 | 0.8% |
| China | 59 | 0.3% |
| Estonia | 58 | 0.3% |
| United Arabic Emirates | 57 | 0.3% |
| Vietnam | 54 | 0.3% |
| Montenegro | 53 | 0.3% |
| Indonesia | 48 | 0.3% |
| Philippines | 48 | 0.3% |
| Singapore | 47 | 0.3% |
| India | 44 | 0.2% |
| Latvia | 41 | 0.2% |
| New Zealand | 36 | 0.2% |
| Lithuania | 32 | 0.2% |
| Tanzania, Sri Lanka | 31 | 0.4% |
| Malta | 29 | 0.2% |
| Mexico | 27 | 0.2% |
| Israel | 26 | 0.1% |
| Egypt | 24 | 0.1% |
| Argentina | 23 | 0.1% |
| Slovenia, Ukraine, Morocco | 22 | 0.3% |
| Korea South | 19 | 0.1% |
| Kenya | 18 | 0.1% |
| Romania | 17 | 0.1% |
| Dominican Republic | 15 | 0.1% |
| Albania, Chile | 14 | 0.2% |
| Serbia | 13 | 0.1% |
| Mongolia, Nepal | 12 | 0.2% |
| Slovakia | 11 | 0.1% |
| Ethiopia, Gambia | 10 | 0.4% |
| Bosnia Herzegovinia, Angola, Zambia, Georgia, Taiwan, Malaysia, Maldives, Costa Rica, Peru | 9 | 0.9% |
| Cabe Verde, Iran (Islamic Republic), Jordan, Nicaragua, Panama, Trinidad and Tobago | 8 | 0.3% |
| Luxembourg, Former Yougoslav Republic of Macedonia, Ghana, Congo , Mauritius, Seychelles, Myanmar, Cambodia, Lebanon | 7 | 0.4% |
| Namibia, Mozambique, Tunisia | 6 | 0.1% |
| Uganda, Iraq, Palestinian State, Bahamas, Colombia | 5 | 0.1% |
| Andorra, Belarus, Armenia, Bhutan, Kazaksthan, Barbados, Jamaica, Ecuador | 4 | 0.2% |
| Kosovo, Algeria, Cameroon, Malawi, Senegal, Somalia, Laos, Oman, Belize | 3 | 0.2% |
| Botswana, Cote d’Ivoire, Gabon, Nigeria, Rwanda, Sierra Leone, Bahrain, Uzbekistan, Antigua and Barbuda, Haiti, Bolivia, Venezuela | 2 | 0.1% |
| Monaco, Moldova, Burundi, Benin, Djibouti, Congo (Kinshasa), Madagascar, Mali, Central African Republic, Afghanistan, Azerbaijan, Brunei Darussalam, Kyrgyzstan, Qatar, Saudi Arabia, Tajikstan, Dominica, Grenada, Guatemala, Honduras, Saint Kitts and Nevis, Saint Lucia, Paraguay, Surinam, Uruguay | 1 | 0.1% |
