# Supplementary material for: Non-prescription purchase of antibiotics during travel abroad among a general adult population in Norway: Findings from the seventh Tromsø Study
Source: PLoS One. 2020 Feb 13;15(2):e0228792. doi: 10.1371/journal.pone.0228792 (PMC7017991; doi:10.1371/journal.pone.0228792)
Supplement: S2 Table — (DOCX) [file pone.0228792.s002.docx]

**Supplementary table 2.**

| **Characteristics** | **n=410** | **OR** | **95% CI** | **p-**  **value** | **AOR** | **95% CI** | **p-**  **value** | ***Deno-***  ***min.*** † |
| --- | --- | --- | --- | --- | --- | --- | --- | --- |
| **Age** (continuous variable) | NA | 1.00 | 0.99-1.01 | 0.853 | 0.99 | 0.98-1.00 | 0.144 | *11001* |
| **Sex** |  |  |  |  |  |  |  |  |
| Men | 3.6% | 1.00 |  | 0.461 | 1.00 |  | 0.986 | *5375* |
| Women | 3.9% | 1.08 | 0.88-1.31 |  | 1.00 | 0.81-1.24 |  | *5626* |
| **Education** |  |  |  |  |  |  |  |  |
| <College/university degree | 3.8% | 1.00 |  | 0.622 | 1.00 |  | 0.313 | *4736* |
| >College/university degree | 3.7% | 0.95 | 0.78-1.16 |  | 0.89 | 0.72-1.11 |  | *6144* |
| **Household income** |  |  |  |  |  |  |  |  |
| Higher | 3.2% | 1.00 |  | 0.001 | 1.00 |  | 0.002 | *6087* |
| Lower | 4.4% | 1.39 | 1.14-1.70 |  | 1.41 | 1.14-1.76 |  | *4604* |
| **Current daily smoking** |  |  |  |  |  |  |  |  |
| No | 3.8% | 1.00 |  | 0.679 | 1.00 |  | 0.639 | *9700* |
| Yes | 3.5% | 0.93 | 0.68-1.30 |  | 0.92 | 0.66-1.29 |  | *1220* |
| **Alcohol intake** |  |  |  |  |  |  |  |  |
| < 2-3 times a week | 3.7% | 1.00 |  | 0.669 | 1.00 |  | 0.694 | *6974* |
| > 2 times a week | 3.8% | 1.05 | 0.85-1.28 |  | 1.04 | 0.84-1.30 |  | *3978* |
| **Childhood mostly lived abroad** |  |  |  |  |  |  |  |  |
| No | 3.6% | 1.00 |  | 0.001 | 1.00 |  | 0.033 | *10265* |
| Yes | 6.1% | 1.77 | 1.27-2.45 |  | 1.47 | 1.03-2.08 |  | *703* |
| **Hospital admission past 12 m** |  |  |  |  |  |  |  |  |
| No | 3.5% | 1.00 |  | 0.001 | 1.00 |  | 0.008 | *9883* |
| Yes | 5.6% | 1.63 | 1.22-2.16 |  | 1.50 | 1.11-2.02 |  | *1036* |
| **Chronic lung disease** ^a^ |  |  |  |  |  |  |  |  |
| No | 3.6% | 1.00 |  | 0.008 | 1.00 |  | 0.055 | *10509* |
| Yes | 7.4% | 2.12 | 1.22-3.68 |  | 1.78 | 0.99-3.22 |  | *190* |
| **Number of travels past 12 m** |  |  |  |  |  |  |  |  |
| 1 travel | 2.7% | 1.00 |  | <0.001 | 1.00 |  | <0.001 | *6675* |
| 2 travels | 4.6% | 1.72 | 1.37-2.17 |  | 1.77 | 1.39-2.24 |  | *2841* |
| 3 travels | 5.9% | 2.26 | 1.65-3.08 |  | 2.44 | 1.78-3.34 |  | *930* |
| 4 travels | 7.9% | 3.08 | 2.00-4.76 |  | 3.18 | 2.02-5.02 |  | *316* |
| > 5 travels | 7.9% | 3.10 | 1.90-5.07 |  | 3.31 | 2.00-5.47 |  | *239* |
| **Diarrhoea during travel past 12m** |  |  |  |  |  |  |  |  |
| No | 3.3% | 1.00 |  | <0.001 | 1.00 |  | <0.001 | *10141* |
| Yes, during one travel | 8.5% | 2.70 | 2.02-3.61 |  | 2.49 | 1.84-3.38 |  | *682* |
| Yes, during two or more travels | 13.5% | 4.53 | 2.55-8.03 |  | 2.64 | 1.43-4.87 |  | *104* |
| **Antibiotic use past 12 months**^b^ |  |  |  |  |  |  |  |  |
| No | 3.1% | 1.00 |  | <0.001 | 1.00 |  | <0.001 | *8696* |
| Yes | 6.1% | 2.04 | 1.66-2.52 |  | 2.08 | 1.67-2.59 |  | *2305* |

OR, odds ratio; CI, confidence interval; AOR, adjusted odds ratio; NA, not applicable; m, months

AORs were adjusted for age, sex, household income, childhood mostly lived abroad, hospital admission past 12 months, number of travel episodes the past 12 months, and antibiotic use in Norway the past 12 months.

*Purchase of antibiotics abroad both *with and without prescription* the past 12 months

†Denominators refer to number of participants included in the multivariable regression analyses of each independent variable (i.e. household income) or for a subgroup of each variable (i.e. 2 travels) and may vary due to missing information.

^a^ Chronic bronchitis/emphysema/COPD

^b^ Data from the Norwegian Prescription Database
